# The gene *MAB_2362* is responsible for intrinsic resistance to various drugs and virulence in *Mycobacterium abscessus* by regulating cell division

**DOI:** 10.1128/aac.00433-24

**Published:** 2024-12-19

**Authors:** Yanan Ju, Lijie Li, Jingran Zhang, Buhari Yusuf, Sanshan Zeng, Cuiting Fang, Xirong Tian, Xingli Han, Jie Ding, Han Zhang, Wanli Ma, Shuai Wang, Xinwen Chen, Tianyu Zhang

**Affiliations:** 1 School of Basic Medical Sciences, Division of Life Science and Medicine, University of Science and Technology of China 12652 , Hefei, China; 2 Institute of Drug Discovery, State Key Laboratory of Respiratory Disease, Guangzhou Institutes of Biomedicine and Health, Chinese Academy of Sciences 74627 , Guangzhou, China; 3 China-New Zealand Joint Laboratory on Biomedicine and Health, Guangzhou Institutes of Biomedicine and Health, Chinese Academy of Sciences 74627 , Guangzhou, China; 4 Institute of Physical Science and Information Technology, Anhui University 12487 , Hefei, China; 5 Guangzhou National Laboratory 612039 , Guangzhou, China; St. George's, University of London, London, United Kingdom

**Keywords:** *Mycobacterium abscessus*, intrinsic resistance, MAB_2362, virulence, cell division

## Abstract

*Mycobacterium abscessus* exhibits intrinsic resistance to most antibiotics, hence leading to infections that are difficult to treat. To address this issue, the identification of new molecular targets is essential for the development or repositioning of therapeutic agents. This study demonstrated that the *MAB_2362*-knockout strain, Mab^Δ2362^, became significantly susceptible to a range of antibiotics, not only *in vitro* but also exhibited susceptibility to rifabutin, bedaquiline, and linezolid *in vivo*. While the bacterial burden of the wild-type *M. abscessus* (Mab^Wt^) increased by over 1 log_10_ CFU/lung in a murine infection model 16 days post-infection, that of Mab^Δ2362^ strain decreased by more than 1 log_10_ CFU/lung, which suggests that the disruption leads to attenuation. Bioinformatics analysis revealed that MAB_2362 shares the highest similarity (41.35%) with SteA, a protein known to influence cell division in *Corynebacterium glutamicum*, suggesting that MAB_2362 might be involved in cell division. Mab^Δ2362^ cells exhibited a median length of 2.62 µm, which was substantially longer than the 1.44 µm recorded for Mab^Wt^ cells. Additionally, multiple cell division septa were observed in 42% of Mab^Δ2362^ cells, whereas none were seen in Mab^Wt^ cells. An ethidium bromide uptake assay further suggested a higher cell envelope permeability in Mab^Δ2362^ compared to Mab^Wt^. Collectively, these findings underscore the role of *MAB_2362* in intrinsic resistance and virulence of *M. abscessus* possibly through the regulation of cell division. Thus, MAB_2362 emerges as a promising candidate for targeted interventions in the pursuit of novel antimicrobials against *M. abscessus*.

## INTRODUCTION

The prevalence of diseases caused by the rapidly growing non-tuberculous mycobacteria (NTM) is increasing (1). *Mycobacterium abscessus*, a serious NTM, has attracted a considerable attention from the global public health community (2–4). The most notable characteristic of *M. abscessus* is its intrinsic drug resistance, posing a substantial threat to global public health (5, 6). Despite long-term treatments involving oral macrolide antibiotics (e.g., azithromycin or clarithromycin [CLR]) and various injectables (e.g., amikacin, cefoxitin, imipenem, and tigecycline), the cure rate remains very low (7). Owing to the intrinsic antibiotic resistance of *M. abscessus*, treatment outcomes for individuals with *M. abscessus* infections are typically unfavorable. Only 30–50% of cases respond effectively to existing treatments, and many patients necessitate surgical resection of lung tissue to manage the infection (8). Surprisingly, while numerous compounds are currently undergoing clinical trials for *M. tuberculosis*, only a few are being tested for *M. abscessus* but with poor activities (4). It is widely acknowledged that the development of novel antimicrobial drugs necessitates a complex and lengthy validation process. Repurposing existing drugs could potentially offer a viable alternative for developing a new treatment strategy. Despite its critical importance in medicine and biology, the intrinsic resistance mechanisms of *M. abscessus* remain largely unexplored. A comprehensive understanding of the intrinsic resistance will contribute not only to elucidating the intricacies of *M. abscessus* physiological mechanisms but also to the identification of potential drug targets.

Virulence-directed therapy is an innovative strategy for combating infections, focusing on neutralizing the pathogenic abilities of microorganisms. This approach prioritizes the disruption of virulence factors to effectively manage infections. Identifying key factors involved in the virulence of *M. abscessus* and discovering drugs that can disrupt these virulence factors could be a promising strategy. Several genes implicated in virulence of *M. abscessus* have been reported. For example, MmpL class transport proteins are abundant in *M. abscessus*. Studies indicate that disrupting the gene encoding MmpL8 significantly reduces the transport efficiency of Glycosyl diacyl-nonadecaol (9), which is a recently identified glycolipid that enhances bacterial adhesion to macrophages and can induce phagosome rupture in macrophages. This mechanism results in the attenuation of mycobacterial virulence (10). Moreover, *M. abscessus* colonies can be differentiated into smooth type (S-type) and rough type (R-type), each displaying distinctive virulence patterns (11). The transition from S-type to R-type results from the mutations in genes in the glycopeptidolipid (GPL) locus, typically associated with the synthesis/secretion of glycopeptidolipids (12, 13). *M. abscessus* exhibits different interactions with immune cells between S-type and R-type. In the S-type state, it mainly exists in a single rod form, while the R-type tends to form clumps that are difficult for phagocytic cells to clear (14). Within macrophages, the R-type resists lysosomal decomposition, enabling rapid and extensive proliferation within the cell (15).

Mycobacterial survival and reproduction are influenced by various factors, with cell division being crucial in their life cycle (16, 17). Mycobacterial cell division is a complex process that includes essential stages like cell wall synthesis, regulation of division proteins, and modulation of both intra- and extracellular environments. These stages significantly impact the virulence and drug sensitivity of the bacteria (18–20). Anomalies in the cell division process of mycobacteria can result in irregular cell morphology and a decreased growth rate, consequently impairing their ability to infect the host (21). Several studies have indicated that the disruption of genes associated with cell division, like *ftsZ* and *ftsW*, can significantly diminish the virulence of *Mycobacterium tuberculosis* (22). Moreover, abnormal mycobacterial division may damage the cell wall structure or destabilize both the intracellular and extracellular environments, increasing the bacteria’s vulnerability to antibiotics (23). Deletion of *MSMEG_0311*, a gene implicated in cell wall synthesis in *M. smegmatis*, results in growth inhibition, colony morphology variations, and altered sensitivity to vancomycin and bedaquiline (BDQ) *in vitro* (24). Hence, factors that influence cell division could play a crucial role in mycobacterial pathogenesis and represent key targets for the development of innovative therapeutic agents. Cell division inhibitors have been reported to exhibit bactericidal activity. For example, cell division inhibitors, which demonstrate an efficacy comparable to that of isoniazid in the acute murine model of *M. tuberculosis* infection, have been investigated (25). Compound T0349 inhibits cell division by specifically binding to SepF protein, thereby blocking its interaction with FtsZ and demonstrating potent inhibitory effects on *M. tuberculosis* (26).

After analyzing the Tn-seq results from previous investigations (27), we identified a correlation between *MAB_2362* and drug susceptibility in *M. abscessus*. Notably, in cultures with sub-inhibitory concentrations of CLR, there was a marked reduction in transposon insertions within the *MAB_2362* gene in the transposon mutant library, indicating that this gene may play an important role in intrinsic drug resistance in *M. abscessus*.

This study reveals the significant role of the *MAB_2362* gene in *M. abscessus*, which regulates cell division and influences the bacterium’s intrinsic resistance and virulence. The results show that after the knockout of the *MAB_2362* gene, *M. abscessus* becomes significantly more sensitive to various antibiotics and exhibits reduced virulence in a mouse infection model. Further studies demonstrate that the deletion of the *MAB_2362* gene leads to increased bacterial cell envelope permeability and enhanced sensitivity to different stresses, which may be one of the reasons for the decreased resistance and virulence. These findings provide important theoretical foundations and potential targets for the development of new antimicrobial drugs against *M. abscessus*.

## RESULTS

### Knockout of *MAB_2362* decreased the intracellular survival of *M. abscessus*

**Fig 1:**
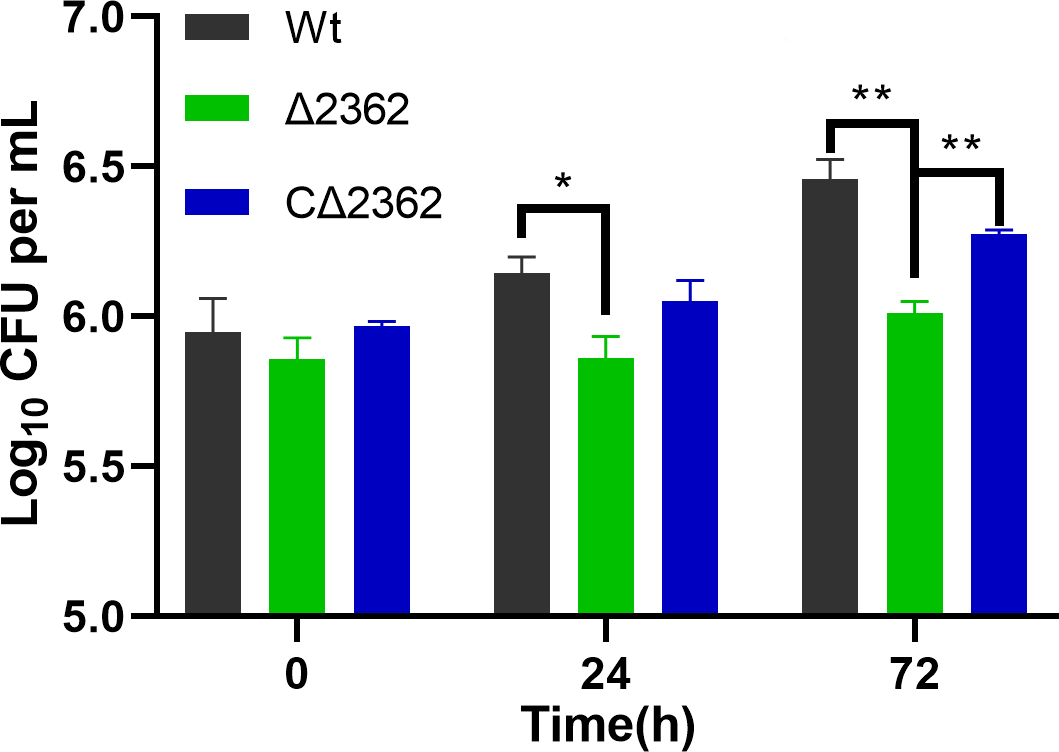
Determination of *MAB_2362*’s effect on *M. abscessus* intracellular survival in macrophages. *MAB_2362* is crucial for intracellular survival of *M. abscessus*. CFU/mL of culture was compared at 0, 24, and 72 hours of post-infection to calculate the intracellular bacteria. The results were representative of mean ± SD of three independent experiments where ^*^ means *P* < 0.05, ^**^ means *P* < 0.01. Wt, wild-type *M. abscessus*; ∆2362, *MAB_2362* knockout strain; C∆2362, *MAB_2362* complemented strain.

We noticed a significant decrease in the frequency of transposon insertion in certain genes within the transposon mutation library under cultivation conditions containing sub-inhibitory concentrations of CLR (27). Therefore, this study utilized CRISPR-assisted non-homologous end-joining (NHEJ) technology (28) to knock out several genes that may be related to intrinsic drug resistance in *M. abscessus* (Table S1). We performed a knockout on *MAB_2362*, deleting 5 base pairs, which resulted in a frameshift mutation near 5′ of the gene (Fig. S1), which was verified by PCR and Sanger DNA sequencing. To explore the potential role of the *MAB_236*2 gene in the interaction between *M. abscessus* and host cells, particularly in terms of intracellular survival, we first conducted experiments using the Raw264.7 macrophage model to evaluate the performance of Mab^Δ2362^ (the knockout strain), Mab^CΔ2362^ (the complemented strain), and wild-type M. abscessus (Mab^Wt^) strains in these aspects. Intracellular bacteria were collected at 0, 24, and 72 hours post-infection, and CFUs were determined by plating different dilutions of lysed cells on agar plates. At the initial infection time point (0 hours), the bacterial loads for all three strains were similar (Fig. 1). By 24 hours, a significant difference was observed between the CFU of Mab^Wt^ and Mab^Δ2362^ strains within Raw264.7 cells (*P* < 0.05), with values of 6.14 ± 0.05 log_10_ CFU/mL and 5.86 ± 0.07 log_10_ CFU/mL, respectively (Fig. 4). From 24 to 72 hours, the intracellular CFUs of all strains showed an increasing trend, and a significant difference was found between the CFUs of Mab^Wt^ and Mab^Δ2362^ strains within Raw264.7 cells (*P* < 0.01), with values of 6.45 ± 0.07 log_10_ CFU/mL and 6.01 ± 0.04 log_10_ CFU/mL, respectively (Fig. 1). A significant difference was also noted between Mab^Δ2362^ and Mab^CΔ2362^ (*P* < 0.01). These findings indicate that *MAB_2362* is crucial for the survival of *M. abscessus* within macrophages.

### Mab^Δ2362^ demonstrated heightened sensitivity to many antibiotics *in vitro*

**Fig 2:**
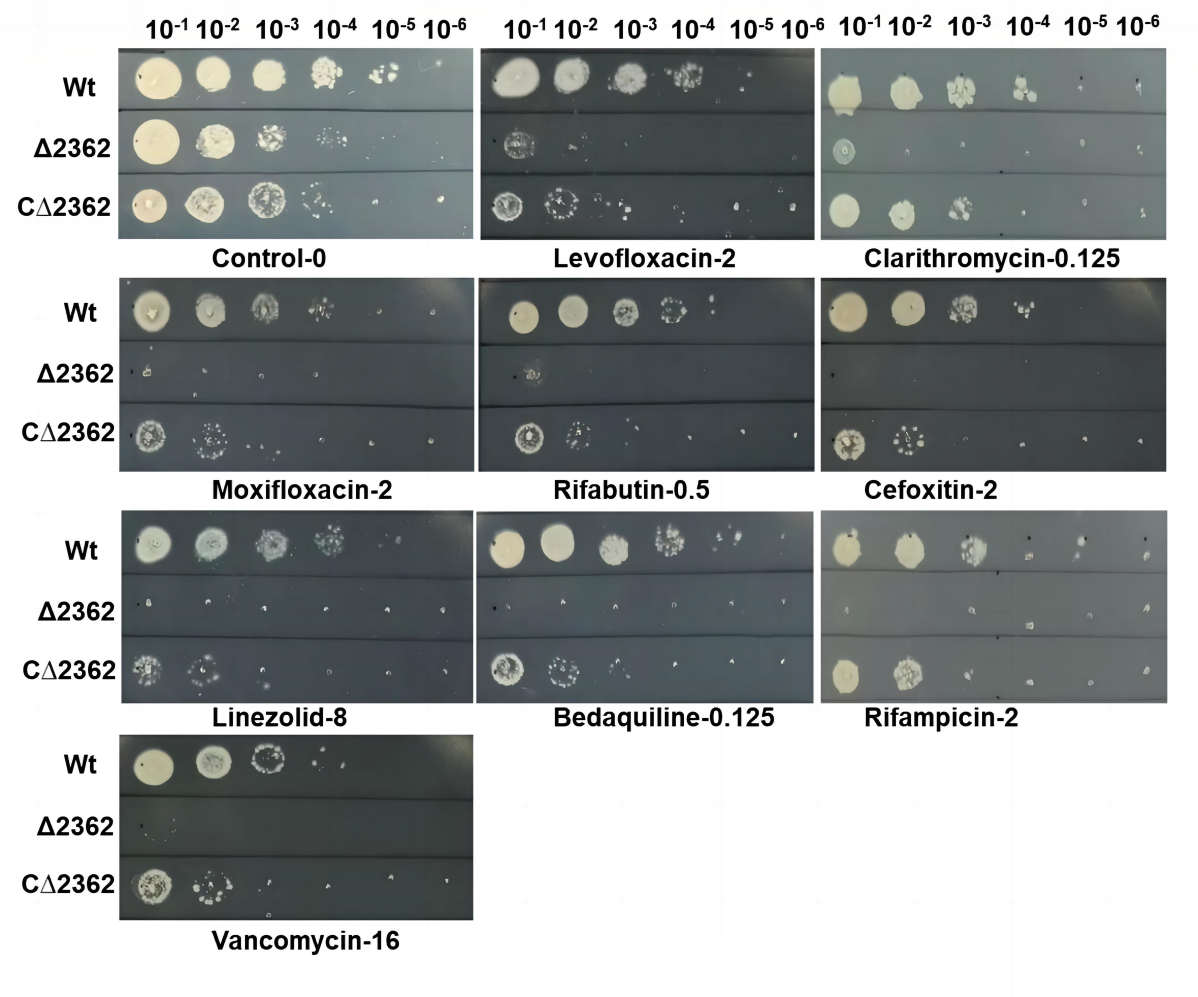
Susceptibility of different *M. abscessus* strains to various antibiotics. Strains with an OD_600_ of 0.6 were serially diluted 10-fold and then spotted onto agar plates supplemented with antibiotics. The plates were then incubated at 37°C for 3 days prior to observation. The unit of drug concentration used in this study is µg/mL. Wt, wild-type *M. abscessus*; ∆2362, *MAB_2362* knockout strain; C∆2362, *MAB_2362* complemented strain.

To further study the role of *MAB_2362* in drug resistance, we tested the susceptibility of different strains to various antibiotics. Mab^Δ2362^ exhibited a marked increase in susceptibility to various antibiotics compared to Mab^Wt^ (Table 1). Notably, the minimum inhibitory concentrations (MICs) for vancomycin, linezolid (LZD), moxifloxacin (MXF), rifampicin, levofloxacin, and CLR were reduced to between 1/64 and 1/16 of that to Mab^Wt^, while other antibiotics showed reductions in MICs ranging from 1/8 to 1/2 of MICs to Mab^Wt^. Complementation of *MAB_2362* gene partially restored resistance in Mab^Δ2362^. As expected, the increased drug susceptibility of Mab^Δ2362^ was clearly evident on solid media plates (Fig. 2). These results strongly indicate that *MAB_2362* plays a significant role in the intrinsic drug resistance of *M. abscessus*. Figure S2 shows that Mab^Wt^, Mab^CΔ2362^, and Mab^Δ2362^ reached a similar growth peak at 72 hours, indicating that the knockout of *MAB_2362* has minimal effect on growth rate. The observed changes in drug susceptibility were not attributable to growth defects.

**TABLE 1:** MICs of various antibiotics against different *M. abscessus* strains

Antibiotic^*b*^	MIC (µg/mL) for strain^*a*^		
	Wt	∆2362	C∆2362
LZD	64	1	32
Vancomycin	128	2	16
MXF	16	0.5	4
Rifampicin	64	2	16
Levofloxacin	32	2	4
Clarithromycin	32	2	8
Cefoxitin	32	4	16
RFB	2	0.25	1
BDQ	0.25	0.0625	0.25

^*a*^ Broth microdilution method was used to determine the MICs. The experiment was performed in triplicate and repeated twice.

^*b*^ Antibiotics for *in vivo* study are underlined. Wt, wild-type *M. abscessus*; ∆2362, *MAB_2362* knockout strain; C∆2362, *MAB_2362* complemented strain.

### Mab^Δ2362^ demonstrated susceptibility to RFB, BDQ, and LZD *in vivo*

**Fig 3:**
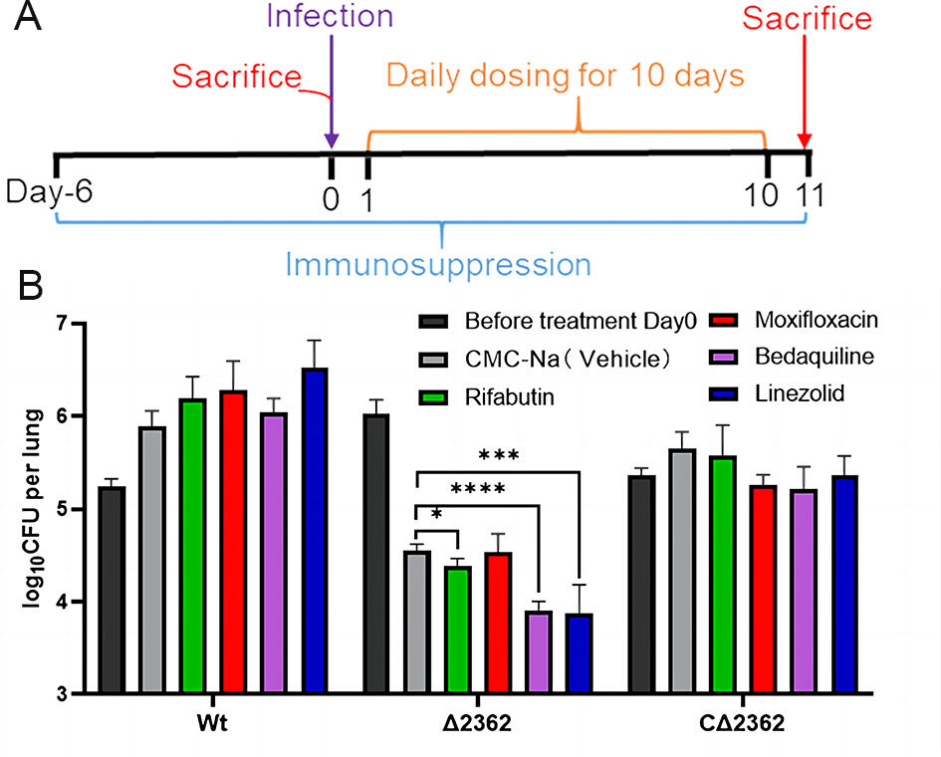
Assessment of susceptibility of different *M. abscessus* strains to drugs *in vivo*. (**A**) Schematic of the *M. abscessus* infection model. Immunosuppression was started 6 days before infection, the mice were sacrificed at the indicated time points to determine lung bacterial burden by CFU enumeration. (**B**) Lung bacterial burden over the 10-day treatment period. Drug doses (mg/kg): RFB 10, LZD 100, MOX 100, and BDQ 20. ^*^ *P* < 0.05; ^***^, *P* < 0.001; ^****^, *P* < 0.0001. Wt, wild-type *M. abscessus*; ∆2362, *MAB_2362* knockout strain; C∆2362, *MAB_2362* complemented strain.

Based on the observations above, *MAB_2362* may play a significant role in the intrinsic drug resistance of *M. abscessus*, and it is speculated that its functional loss might affect the drug sensitivity of the bacterium *in vivo*. Therefore, we tested the *in vivo* susceptibility of the Mab^Δ2362^ strain to various antibiotics with promising MICs by enumerating the bacterial colony-forming units (CFUs) in the lungs of infected mice following 10-day treatment with rifabutin (RFB), MXF, BDQ, or LZD. As shown in Fig. 3, both BDQ (*P* < 0.0001) and LZD (*P* < 0.001) significantly reduced the Mab^Δ2362^ burden in mouse lungs compared to the untreated control group. However, there was no significant difference in the CFUs of the Mab^Wt^ strain and the Mab^CΔ2362^ strain in the lungs of mice after drug treatment compared to the untreated controls. Additionally, although the difference in bacterial load between the RFB treatment group and the solvent group appeared smaller, (4.37 ± 0.08 log_10_ CFU/lung vs 4.54 ± 0.09 log_10_ CFU/lung), it was statistically significant (*P* < 0.05). Despite the significantly low MIC of MXF against Mab^Δ2362^ (Table 1), it failed to exhibit substantial *in vivo* activity when compared to untreated controls in both Mab^Δ2362^- and Mab^C∆*2362*^-infected mice, which parallels the response observed in MXF-sensitive *M. tuberculosis* H37Rv in the persistent status (29). It is also notable that the drug-treated group even showed a slightly elevated lung bacterial burden compared to that of the untreated group in Mab^Wt^-infected mice, though this was not statistically significant. Thus, RFB, BDQ, and LZD were active against Mab^∆2362^ *in vivo* at the tested doses.

### Mab^Δ2362^ exhibited attenuation in the mouse model

**Fig 4:**
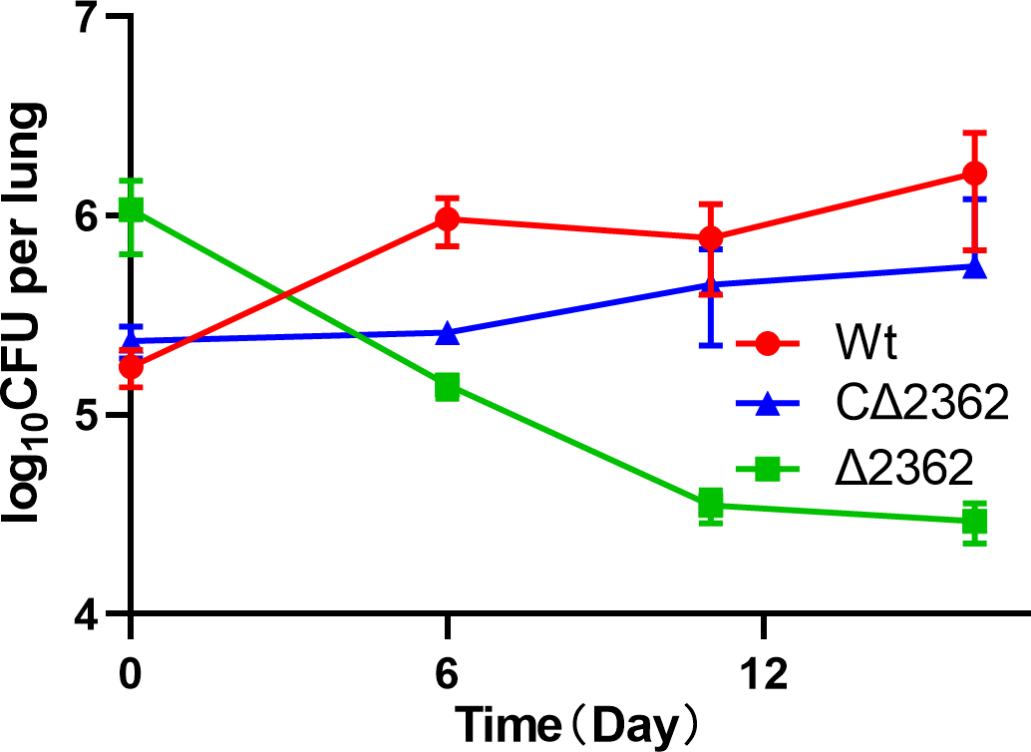
The virulence assessment of different *M. abscessus* strains. All the mice started continuous immunosuppression with dexamethasone 1 week before infection. Five mice per group at each time point were sacrificed on days 0, 6, 11, and 16 separately for CFU enumeration to determine the bacterial burden in lungs. Wt, wild-type *M. abscessus*; ∆2362, *MAB_2362* knockout strain; C∆2362, *MAB_2362* complemented strain.

On the day of infection (Day 0), the mean (± standard deviation [SD]) lung CFU counts in mice infected with Mab^∆2362^ were 6.06 ± 0.13 log_10_. By Day 16, the burden of Mab^Δ2362^ decreased to 4.50 ± 0.05 log_10_ CFU/lung (Fig. 4). In contrast, the bacterial burdens of both Mab^C∆2362^ and Mab^Wt^ increased from 5.37 ± 0.08 log_10_ CFU/lung to 5.54 ± 0.47 log_10_ CFU/lung, and from 5.24 ± 0.10 log_10_ CFU/lung to 6.16 ± 0.26 log_10_ CFU/lung, respectively. The trends for progressive increase/decrease in bacterial burden for all three strains were consistent throughout the experiment. In the mouse model, the lung bacterial burden of the Mab^Δ2362^ strain decreased significantly over time, while the bacterial burden of the Mab^Wt^ and Mab^CΔ2362^ increased. These results indicate that the knockout of the *MAB_2362* gene leads to a significant reduction in the virulence of *M. abscessus*.

### Mab^Δ2362^ showed an increase in cell length and the formation of multiple septa

**Fig 5:**
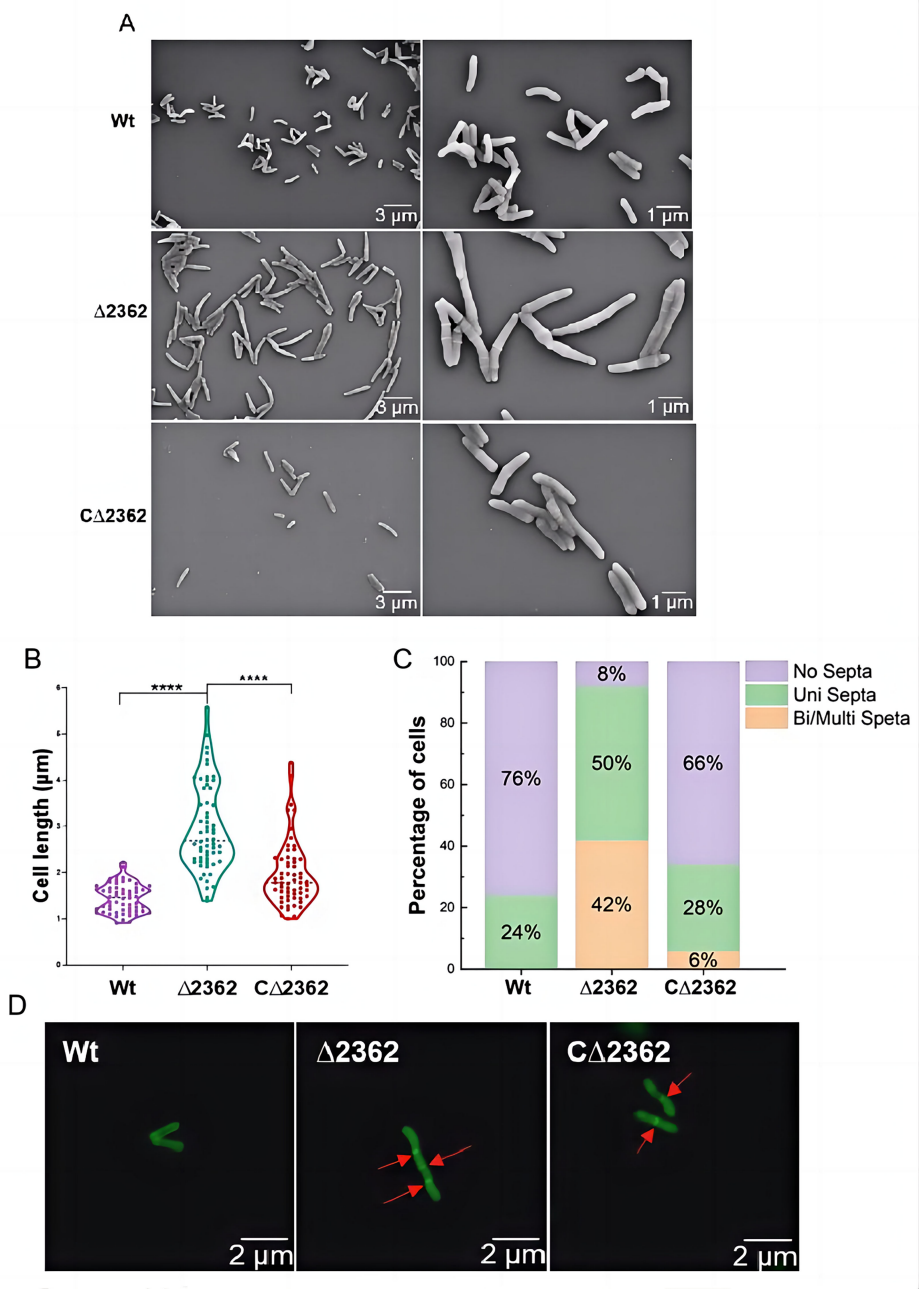
Microscopic phenotypes of different *M. abscessus* strains. (**A**) Representative micrographs of the different strains examined by cryo-scanning electron microscope microscopy. (**B**) Violin plots comparing the cell lengths of the three strains. Statistical significance was established using the one-way analysis of variance test. ^****^, *P* < 0.0001. (**C**) A bar graph shows the proportion of cells containing different numbers of septa among the three strains. (**D**) Images of the different strains obtained by laser confocal microscopy. The cells were labeled with HADA for 4 hours. Red arrows indicate the cellular septa. Wt, wild-type *M. abscessus*; ∆2362, *MAB_2362* knockout strain; C∆2362, *MAB_2362* complemented strain.

Bioinformatics analysis indicated that MAB_2362 exhibits the highest similarity (41.35%) to SteA (Fig. S3). MAB_2362 is annotated as a putative cytokinetic ring protein SteA (30), which is known to affect cell division in *Corynebacterium glutamicum*. This suggests that MAB_2362 may also play a role in cell division. Consequently, the Cryo-SEM analysis was conducted to assess the impact of *MAB_2362* on cell division. Notably, a significant increase in cell length was observed for Mab^∆2362^ compared to Mab^Wt^ (Fig. 5A). Based on the analysis of randomly selected 50 cells, the median cell length of Mab^Wt^ was 1.44 µm, while the median cell length of Mab^∆2362^ reached 2.62 µm. The knockout of *MAB_2362* was partially compensated by *MAB_2362* under the strong promoter *hsp60*, resulting in a median cell length of 1.78 µm (Fig. 5B). To quantify the septation patterns, 50 randomly selected cells from each strain were counted. 42% Mab^∆2362^ cells showed bi- or multiseptate phenotype, while all the Mab^Wt^ cells had zero or only one septum (Fig. 5C). In the case of the complemented strain Mab^C∆2362^, the proportion of cells with bi- or multiseptate phenotype was 6%. The visualization of septa per cell was also achieved using a fluorescent HCC-amino-D-alanine hydrochloride (HADA) (31), which labels septa and other sites of new cell wall synthesis. The HADA fluorescence analysis revealed the typical presence of 0 or 1 septum in Mab^Wt^ or Mab^C∆2*362*^ and 3 septa in Mab^∆2362^ (Fig. 5D). These results indicate that the *MAB_2362* gene plays a crucial role in cell division.

### Mab^∆2362^ showed increased cell envelope permeability

**Fig 6:**
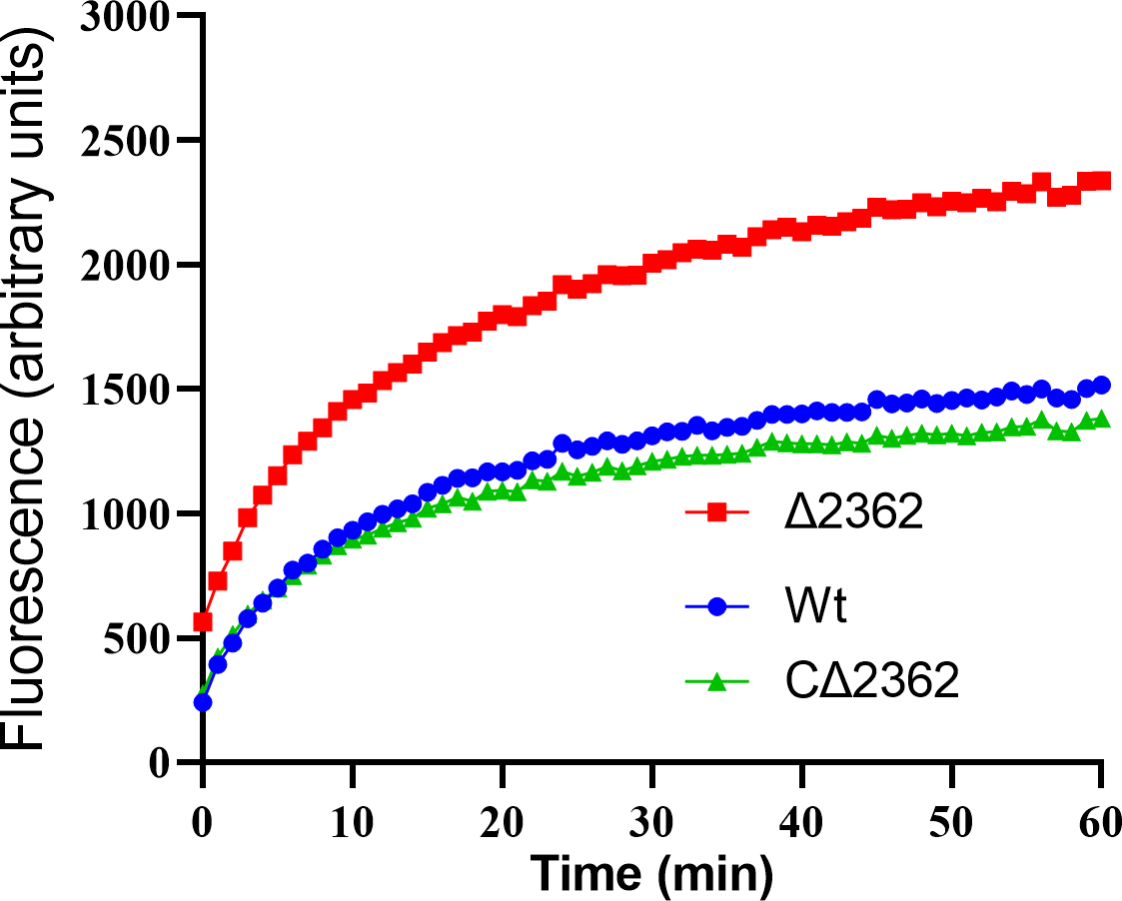
The cell envelope permeability of different *M. abscessus* strains. The increase in fluorescence intensity indicates an accumulation of EtBr. Wt, wild-type *M. abscessus*; ∆2362, *MAB_2362* knockout strain; C∆2362, *MAB_2362* complemented strain.

Aberration in cell division is often associated with cell envelope permeability (23). Based on this, we postulated that the cell envelope permeability of Mab^Δ2362^ might be heightened. To substantiate this postulation, this study scrutinized the accumulation of ethidium bromide (EtBr) (32) in Mab^∆2362^, Mab^C∆2362^, and Mab^Wt^ (Fig. 6). As expected, Mab^Δ2362^ accumulated a larger amount of EtBr compared to the parental strain Mab^Wt^. On the contrary, the complemented strain Mab^CΔ2362^ accumulated an amount of EtBr similar to that of Mab^Wt^. This result indicated that the knockout of *MAB_2362* results in increased cell envelope permeability in *M. abscessus*.

### *MAB_2362* played a role in the differential stress response of *M. abscessus*

**Fig 7:**
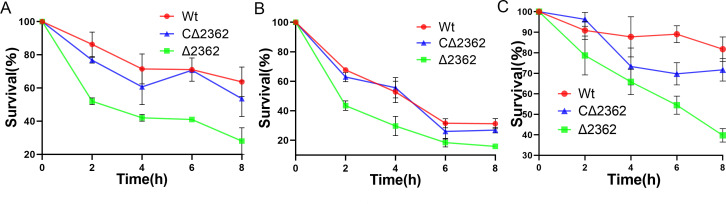
Survival rates of different *M. abscessus* strains under different stress conditions. (**A**) SDS stress. Cultures were grown in the presence of SDS (0.025%) at 37°C. (**B**) Oxidative stress. The cultures were grown in 7H9 medium supplemented with H_2_O_2_ (50 mM). (**C**) Acidic stress. Cultures were exposed to adjusted 7H9 medium pH 4.75 for 2, 4, 6, and 8 hours, respectively. Growth was monitored by CFU counting at 0, 2, 4, 6, and 8 hours. Wt, wild type *M. abscessus*; ∆2362, *MAB_2362* knockout strain; C∆2362, *MAB_2362* complemented strain.

To elucidate the potential mechanisms underlying the decreased virulence observed in the *MAB_2362* knockout strain, we measured the sensitivities of Mab^Wt^, Mab^Δ2362^, and Mab^CΔ2362^ to SDS, H_2_O_2_, and acidic condition. As shown in Fig. 7, under SDS stress (0.025%), the survival rate of the Mab^Δ2362^ strain dropped to approximately 28% after 8 hours compared to 63.67% for Mab^Wt^ and 53.57% for Mab^CΔ2362^. Under oxidative stress (H_2_O_2_ 50 mM), the survival rate of the Mab^Δ2362^ strain plummeted to 15.92%, while the survival rates of the Mab^Wt^ and Mab^CΔ2362^ strains were 31.25% and 26.88%, respectively. In acidic condition (pH 4.75) after 8 hours of incubation, the Mab^Wt^ strain maintained a survival rate of 81.77%; the survival rate of the Mab^Δ2362^ strain declined more rapidly, falling to 39.75%, while the survival rate of the Mab^C∆2362^ strain was 71.67%. These results indicate that *MAB_2362* plays a crucial role in modulating *M. abscessus* tolerance to various stress conditions. Strains lacking the *MAB_2362* gene exhibit significantly lower survival rates under surfactant, oxidative, and acidic stress, indicating the importance of this gene in maintaining bacterial viability under these environmental pressures.

## DISCUSSION

*M. abscessus* exhibits intrinsic resistance to the majority of drugs, leading to a scarcity of clinically efficacious therapeutics (33). The development of novel antimicrobials necessitates significant financial investment and time commitment. Compared to the *de novo* development of drugs, identifying existing medications that target specific genes or proteins of *M. abscessus* but shows no activity when used alone can improve the efficacy of other existing drugs. This approach has the potential to reduce time and cost associated with pharmacokinetic studies and toxicity research (34), offering a relatively safe and efficient approach. This is highly pertinent for the discovery of new anti-*M*. *abscessus* drugs and new therapies. Using transposon mutagenesis technology, it has been discovered that certain genes become essential in the presence of drugs, prompting further characterization (35). This technique is highly effective for identifying potential synthetic lethal drug targets.

A comprehensive understanding of the genetic basis of intrinsic antibiotic resistance and the identification of virulence factors are prerequisites for the discovery and development of synergistic drug combinations. In such combinations, one agent can restore the activity of a failing antibiotic by disrupting one of the aforementioned mechanisms. A notable example is the identification of avibactam, a potent inhibitor of *M. abscessus* β-lactamase, which efficiently enhances the activities of several β-lactams when used in combination against *M. abscessus*, increasing their efficacy by 4- to 32-fold (36, 37). Blocking pathogen virulence mechanisms is a highly effective approach to combating bacterial resistance. To date, several classes of virulence blockers have been developed and are utilized in clinical practice (38–40). Our study revealed that the knockout of *MAB_2362* could enhance the *in vivo* sensitivity of *M. abscessus* to a range of drugs and increase its sensitivity to RFB, BDQ, and LZD, as well as reduce virulence in mice. This indicates that inhibitors of MAB_2362 may have the potential to render *M. abscessus* sensitive to these drugs *in vivo* and/or to the host immune system.

The discrepancy observed in this study between *in vitro* and *in vivo* activities of MXF could possibly be due to MXF only demonstrating bacteriostatic activities, not bactericidal activities. In this *M. abscessus* infection model, Mab^Δ2362^ burden in the lungs decreased continuously even without treatment. Additionally, MXF lacks bactericidal activity against *M. tuberculosis* persisters (29) which is consistent with the enhanced MICs of MFX to non-replicating *M. tuberculosis* in different broth media under low oxygen condition in a recent study (41). Therefore, the status of *M. abscessus* in this mouse model might be close to persistence. Another possibility is that a short-term treatment regimen might not suffice to demonstrate its efficacy (42).

The antibiotic-treated wild-type strain shows better growth than the vehicle control. Our previous research has also noted similar phenomena; for instance, after treatment of mice infected Mab^Wt^ using RFB, we observed a slight increase in bacterial load in the lungs of mice compared to the untreated control group (32). We speculate that these drugs may not only be ineffective against *M. abscessus* infection but could also exert side effects on the mice, potentially contributing to the increased bacterial load.

A previous study found that a higher infection dose of *M. abscessus* could maintain a slow increase or relatively stable lung bacterial burden, while a lower infection dose could lead to a rapid decrease in lung bacterial burden (43). Notably, the amount of the Mab^Δ2362^ strain at the time of infection was higher than that of Mab^Wt^ and Mab^CΔ2362^, yet it exhibited a significantly decreased bacterial burden. Therefore, if the initial amount of Mab^Δ2362^ in the lung were lower than or comparable to that of Mab^Wt^ and Mab^CΔ2362^, the reduction in Mab^Δ2362^ could potentially be even greater than what was observed.

In this study, we found that the complemented strain did not fully restore the phenotype to that of the wild type. To further validate the expression level of *MAB_2362* in the complemented strain, we measured the transcriptional expression levels of *MAB_2362* in different strains using qRT-PCR (Fig. S4). The mRNA expression levels of *MAB_2362* in Mab^Wt^ and Mab^∆2362^ are similar, possibly because the *MAB_2362* gene in Mab^∆2362^ only has a few base mutations, none of which occur in the promoter region, thus having no impact on the transcription of *MAB_2362*. However, the mRNA expression level of *MAB_2362* in Mab^C∆2362^ increased more than 20-fold compared to Mab^Wt^ and Mab^∆2362^. We speculate that this is due to the transformation of Mab^Δ2362^ with an episomal complementing plasmid, which is characterized by high-copy replication, and the expression of *MAB_2362* is controlled by the strong promoter *hsp60*, resulting in higher transcription of mRNA. Interestingly, although the complementation strain has a high *MAB_2362* mRNA level, it does not fully restore the phenotype. In our study, we explored various complementation strategies using single-copy plasmids, multicopy plasmids, natural promoters, and strong promoters. However, these attempts failed to fully restore the complemented strains to a wild-type phenotype (data not shown). Considering the potential polar effects, we also attempted to complement both the *MAB_2362* and *MAB_2363* genes simultaneously. The double-gene complemented strain showed almost no difference in drug sensitivity compared to the strain that had only *MAB_2362* complemented (Table S4; Fig. S5). Additionally, qRT-PCR results indicated that the mRNA expression level of *MAB_2362* in the complemented strains was significantly higher than that in the wild-type strain, suggesting that the partial restoration of the phenotype might not be due to differences in expression levels. We hypothesize that the MAB_2362 protein may play a crucial role in specific stages of cell division. This implies that even though the total protein level increases, failure to correctly localize the protein to its site of action might impede its physiological function (44), or the temporal and spatial sequence of MAB_2362 expression may play a crucial role in its functional execution.

During the cell division process, the synthesis and rearrangement of the cell wall are involved. Abnormal cell division can lead to changes in the structure of the cell wall (45). Increased cell envelope permeability may primarily account for both the virulence attenuation and altered drug susceptibility as observed in a recent cell wall-disrupted-*M. abscessus* due to knockout of *embC* (32). In addition, a previous study has shown that the reduction of cell envelope permeability of *M. tuberculosis* led to increased resistance to low pH, SDS (46), and oxidative stress (47). We speculate that *MAB_2362* might contribute to stress resistance by modulating the permeability of the cell envelope. These findings suggest that *MAB_2362* is related to cell division, impacting the permeability of the mycobacterial cell envelope and other cellular physiological characteristics.

In conclusion, this study demonstrates that the *MAB_2362* gene plays a significant role in regulating cell division in *M. abscessus*. Its deletion leads to division defects and changes in cell morphology, disrupting cell division, compromising cell wall integrity, and increasing permeability. As a result, this affects the intrinsic antibiotic resistance and survival of *M. abscessus* within the host. Therefore, developing inhibitors that specifically target MAB_2362 represents a promising strategy to counteract intrinsic antibiotic resistance, sensitizing *M. abscessus* to existing antibiotics and inhibiting its survival and dissemination within the host.

## MATERIALS AND METHODS

### Strains, plasmids, and experimental conditions

This study utilized CRISPR-assisted NHEJ technology (28) to knock out several genes that may be related to intrinsic drug resistance in *M. abscessus* (Table S1). We performed a knockout on *MAB_2362*, deleting five base pairs, which resulted in a frameshift mutation near 5′ of the gene (Fig. S1), which was verified by PCR and Sanger DNA sequencing. The strain used in this experiment was Mab subsp. *abscessus* GZ002 (NCBI GenBank accession number CP034181), a previously characterized clinical isolate (32, 48, 49). The strain Mab-pNHEJ-Cpf1 (28) for gene knockout was constructed in our laboratory. Both strains were cultured in 7H9 medium (supplemented with 10% OADC and 0.05% Tween 80) or on 7H10 solid medium at 37°C, with the addition of kanamycin (KAN 100 µg/mL) if necessary. *Escherichia coli* DH5α was grown at 37°C in Luria Bertani (LB) broth and on LB agar, supplemented with KAN (50 µg/mL) or zeocin (30 µg/mL) when required. Restriction enzymes *Bpm* I, *Hin*d III, and *Bam*H I were obtained from New England Biolabs, while *Cla* I was sourced from TaKaRa. The plasmids used in this study are listed in Table S2. The plasmid pCR-Zeo was used to express the guide RNA (28). The plasmid pMV261-MAB_2362, designed for overexpressing *MAB_2362*, was constructed using the vector pMV261. The *MAB_2362* gene was amplified from *M. abscessus* and cloned into plasmid pMV261 following digestion with *Bam*H I and *Cla* I. Subsequently, the constructed plasmid was electroporated into the *MAB_2362* knockout strain.

### Knockout and complementation of *MAB_2362*

This study utilized CRISPR-assisted NHEJ to knock out *MAB_2362*. Primers used in this study are listed in Table S2. The guide RNA sequence was incorporated into a pair of primers, which were subsequently annealed and ligated into the linearized pCR-Zeo plasmid. This plasmid was then introduced into Mab-pNHEJ-Cpf1 strain via electroporation. This strain contains the pNHEJ-Cpf1 plasmid, which carries the CRISPR-Cpf1 system—a gene editing tool that can locate and cleave specific DNA sequences. When exogenous DNA is electroporated into these cells, the Cpf1 nuclease, guided by specific crRNA, can accurately induce DNA breaks at the *MAB_2362* gene locus. Subsequently, the NHEJ system attempts to repair the break, which usually results in nucleotide deletion, insertion, or mutation. The NHEJ repair pathway does not require a homologous DNA template, simplifying the process of genome editing (50). The resulting transformants were allowed to grow on 7H11 agar plates supplemented with KAN (100 µg/mL), anhydrotetracycline (200 ng/mL), and zeocin (30 µg/mL) at 30°C for 72 hours. Colonies were screened by PCR and verified by Sanger sequencing performed by Sangon Biotech Co., leading to the successful isolation of the *MAB_2362* knockout strain.

### Intracellular survival assay

Macrophages were seeded at 5 × 10^4^ cells/well in 12-well tissue culture plates for survival rate assay. The cells were infected with *M. abscessus* at MOI of 10:1. After infection for 4 hours, the remaining bacteria outside macrophages were washed three times with Dulbecco’s Modified Eagle Medium (Biotechnology Co., Ltd.). Then 200 µL of medium containing 250-µg/mL amikacin was added and incubated for 2 hours to kill extracellular bacteria. The extracellular bacteria were thoroughly washed away with serum-free medium, and during subsequent culture, amikacin was maintained at 50 µg/mL to inhibit the growth of residual extracellular bacteria. At 0, 24, and 72 hours post-infection, the cells were washed three times with phosphate-buffered saline (PBS), and 200 µL of sterile double-distilled water was added to each well to lyse the cells. After appropriate dilution with PBS, the solution was spread onto antibiotic-free 7H10 solid plates. The CFUs of *M. abscessus* were counted after incubation for 3 days.

### Antibiotic susceptibility assay

*M. abscessus* was cultivated in 7H9 medium until it reached an OD_600_ of 0.6 to 0.8. Subsequently, a 10-fold gradient dilution was performed on spot plates using 7H10 solid medium supplemented with various concentrations of antibiotics. Control plates containing no antibiotics were also prepared. The broth dilution method was employed to determine the MIC. In this assay, cells were inoculated into 7H9 medium at a density of 5 × 10^5^ CFU/mL, along with twofold serial dilutions of the drugs. Incubation periods were 14 days for CLR and 3 days for other drugs, maintained at 37°C. Three internal controls were established for each microwell, and the entire experiment was replicated three times.

### Measurement of sensitivity to SDS, low pH, and oxidative

*M. abscessus* was exposed to three distinct stress conditions: 0.025% SDS, 50 mM H_2_O_2_, and acidic stress (pH 4.75). Each experiment was replicated three times. Different strains were cultured in 7H9 medium to an OD_600_ of 0.8–1.0. After washing twice with 7H9 medium, the OD_600_ was adjusted to 0.5. Culture in media contained 0.025% SDS, 50 mM H_2_O_2_, and pH 4.75, respectively. CFUs were calculated at time points of 0, 2, 4, 6, and 8 hours.

### Mice infection and treatment

All animal procedures were approved by the Institutional Animal Care and Use Committee of the Guangzhou Institutes of Biomedicine and Health (2023064). In this study, female BALB/c mice aged 6–7 weeks and weighing 18–21 g were used to establish the murine infection. Immunosuppression was initiated 1 week before infection with a 4 mg/kg dose of dexamethasone and continued throughout the experiment (51). Dexamethasone was dissolved in PBS, freshly prepared, and administered subcutaneously once daily. The bacterial suspension used for infection was cultured to an OD_600_ of 1.0 to 1.2 and then diluted and inoculated into a suspension with an OD_600_ of 0.1. This suspension was used for mice infection after the bacteria reached the logarithmic growth stage. The mice were infected via inhalation and randomly divided into three groups, each infected with Mab^Wt^, Mab^Δ2362^, and Mab^C∆2362^ strains, respectively. On the day of infection, mice from each group were sacrificed to determine the initial bacterial burden in the lungs. The remaining mice in each group were randomly assigned to receive no antibiotic treatment on the day of infection.

### Antibiotics and treatment regimen

Drug administration was started a day after infection and continued for 10 days. In the treatment regimen for mice, RFB and LZD were dissolved in a solution of 0.4% CMC-Na, and MXF was dissolved in PBS, while BDQ was formulated using a vehicle comprising 20% hydroxypropyl-β-cyclodextrin and 1.5% hydrochloric acid. The dosage (mg/kg) regimen consisted of 10 for RFB, 100 for both LZD and MXF, and 20 for BDQ. Each medication was orally administered once daily.

### Microscope image acquisition

For the determination of cell length and enumeration of septal counts, this investigation employed a cryo-scanning electron microscope (Cryo-SEM, Gemini SEM 300, Carl Zeiss, UK). Bacteria were grown to an OD_600_ of 0.6 to 0.8, after which, 1 mL of the bacterial suspension was centrifuged, followed by fixation with 4% glutaraldehyde for 1 hour at room temperature. After centrifugation, the supernatant was discarded, and the cells were washed twice with PBS before resuspension. A sufficient quantity of the bacteria was transferred onto a cell crawler, dried, and subsequently washed three times with PBS. The cells were then sequentially dehydrated using 70%, 80%, 90%, and 100% ethanol solutions. Drying was achieved via the critical point drying technique. After gold sputtering, cellular imaging was conducted under the microscope at magnifications of 4,000 or 10,000. Cell length measurements were executed using Nano Measurer 1.2, with subsequent image acquisition, while data processing was facilitated by GraphPad Prism 8.3.0. To label peptidoglycan, 1-mM HADA was used to supplement *M. abscessus* cultures. Samples were centrifuged, then washed three times with PBS-0.05% Tween 80, and fixed with 4% paraformaldehyde for 30 minutes prior to microscopic examination. Image analysis was performed using Origin 2021 software. This study utilized Chinese version DNAMAN software (V6) for the analysis of the MAB_2362 sequence.

### EtBr uptake assay

Mab^Wt^, Mab^Δ2362^, and Mab^C∆2362^ were cultivated until the OD_600_ reached 0.6 to 0.8. The bacterial cells were then harvested by centrifugation. The supernatant was discarded, and the pellet was resuspended in PBS containing 0.05% Tween 80 for washing and then diluted to OD_600_ 0.8 in the same solution. EtBr was diluted to a concentration of 4 µg/mL using PBS-0.05% Tween 80, and glucose was added to a final concentration of 0.8%. This mixture, comprising 4 µg/mL EtBr and 0.8% glucose, was transferred to an opaque 96-well plate in volumes of 100 µL, followed by the addition of 100 µL of the prepared bacterial suspension. Subsequently, real-time fluorescence measurements were carried out using a fluorescence spectrometer (PerkinElmer), with excitation and emission wavelengths set at 530 and 595 nm, respectively. The results were normalized against the fluorescence of EtBr. The experiment was conducted in triplicate and was repeated on three separate occasions.

## SUPPLEMENTAL MATERIAL

Supplemental material aac.00433-24-s0001.docx Tables S1 to S4; Fig. S1 to S5.

ASM does not own the copyrights to Supplemental Material that may be linked to, or accessed through, an article. The authors have granted ASM a non-exclusive, world-wide license to publish the Supplemental Material files. Please contact the corresponding author directly for reuse.
