# Supplementary material for: The gene MAB_2362 is responsible for intrinsic resistance to various drugs and virulence in Mycobacterium abscessus by regulating cell division
Source: Antimicrob Agents Chemother. 2024 Dec 19;69(2):e00433-24. doi: 10.1128/aac.00433-24 (PMC11823648; doi:10.1128/aac.00433-24)
Supplement: Supplemental material — Tables S1 to S4; Fig. S1 to S5. [file aac.00433-24-s0001.docx]

## Supplemental Material

**Table S1. Selected genes for knockout**

| **Selected genes** | **Whether the knockout was successful** |
| --- | --- |
| *MAB_0490*  *MAB_2217*  *MAB_2297*  *MAB_3130*  *MAB_2362*  *MAB_4059*  *MAB_4395*  *MAB_4132*  *MAB_3837* | Yes  Yes  Yes  Yes  Yes  Yes  Yes  Yes  No |

**Table S2. Plasmids used in this study**

| **Plasmids** | **Description** |
| --- | --- |
| pMV261  pCR-Zeo  pNHEJ-cpf1  p-MAB_2362 | A plasmid used for complementation, containing kanamycin resistance and the strong promoter *hsp60* from mycobacterium.  Used for expressing crRNA. Zeocin resistance.  Used for gene knockout in *M. abscessus*. kanamycin resistance gene, contains the *cpf1* sequence encoding Cas12a protein induced by dehydrated tetracycline.  The plasmid used for complementation of the *MAB_2362* gene. The *MAB_2362* gene inserted downstream of the *hsp60* promoter in pMV261. |

**Table S3. Primers used in this study**

| **Primers** | **Sequence (5’-3’)** |
| --- | --- |
| cr-2362-F  cr-2362-R  JD-Zeo-R  hb-2362-F  hb-2362-R  pZeo-F  hb-yz-F | ATTCCGGGAACACCATCGAGTTCAA  AGCTTTGAACTCGATGGTGTTCCCGGAATCT  GGTGAATCCTCCTGAATATGTAGAG  GCCAAGACAATTGCGGATCCATGATCACCCC  TATGAACTTGACC  GTTAACTACGTCGACATCGATTCAGCTGACC  AGGTTCTGCAC  ATAACGTTGGCACTCGCGAC  CAGCGAGGACAACTTGAGCC |


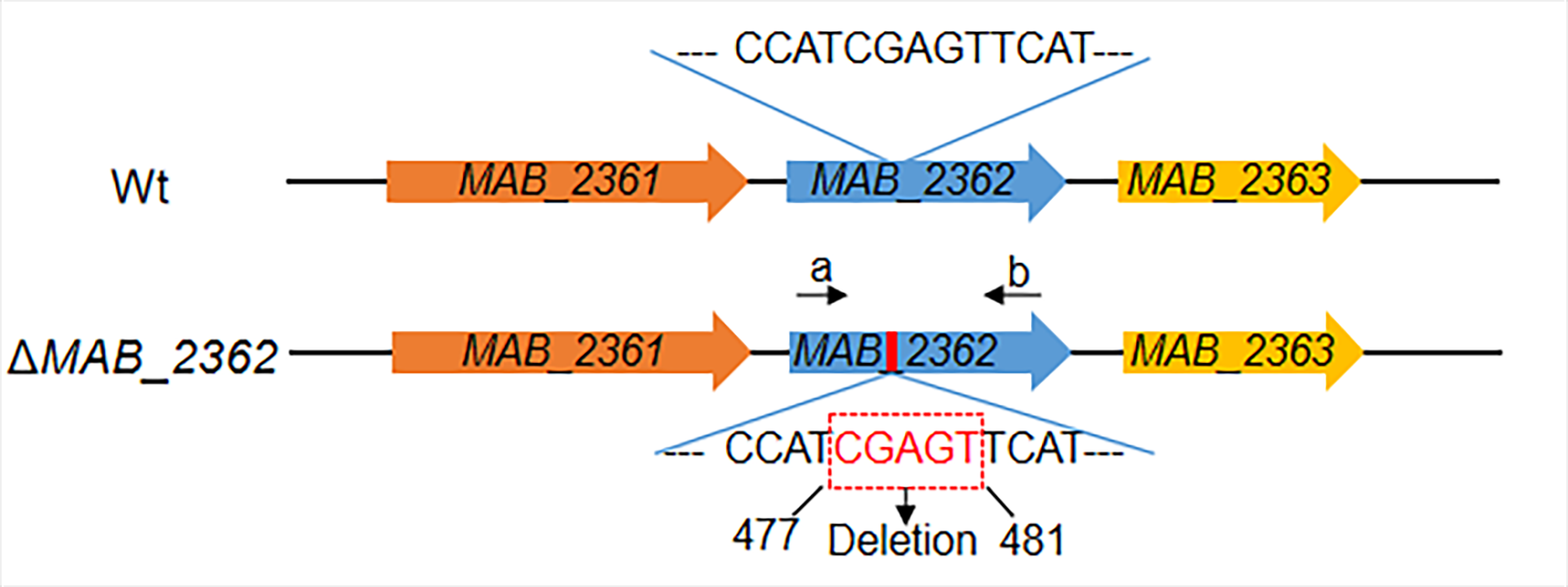
**Figure S1. *MAB_2362* gene specified deletion site diagram.** *MAB_2362* consists of 1197 bases, in which the bases at positions 477-481 have been knocked out, leading to a frameshift mutation and thus obtaining a *MAB_2362* knockout strain. The arrows labeled with a and b indicated the primers (hb-2362-F/ hb-2362-R) used for knockout identification.


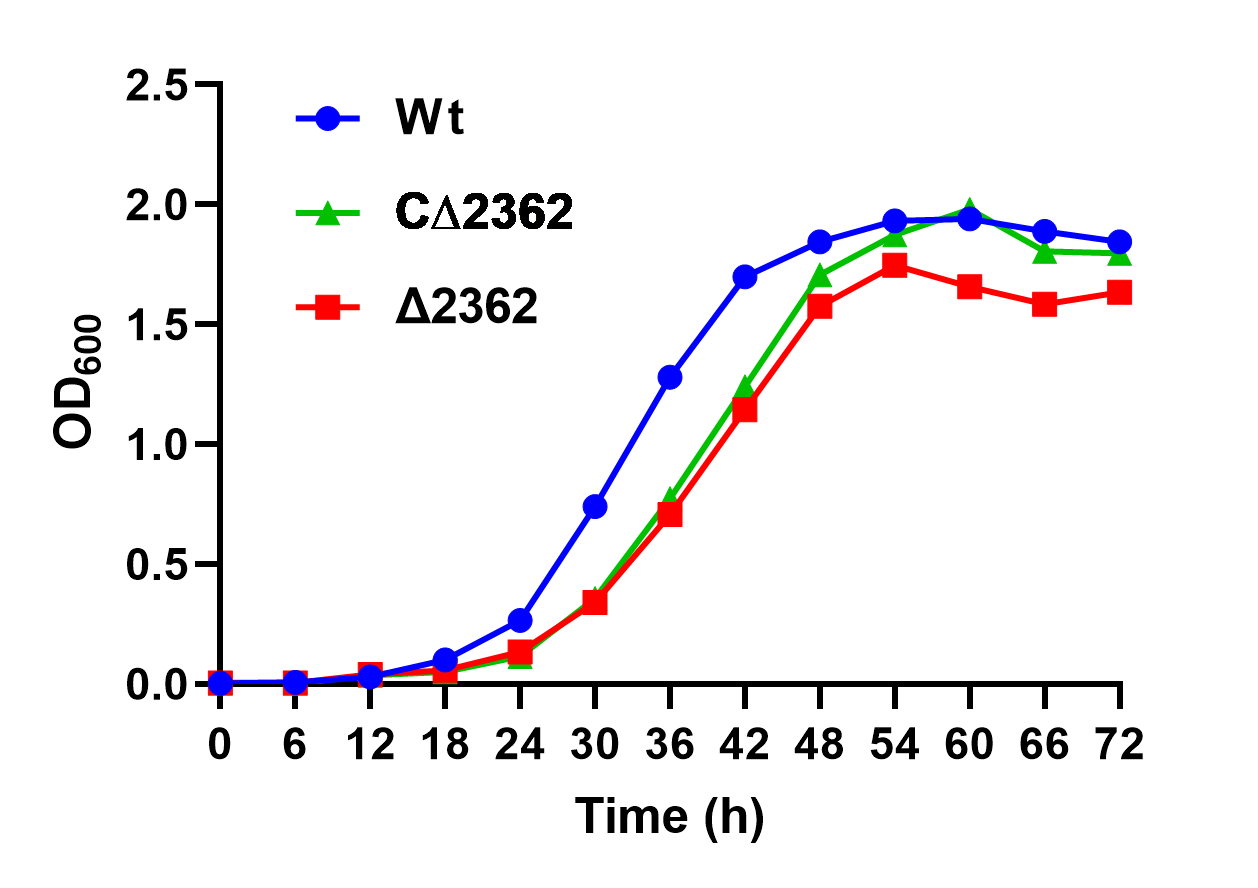
**Figure S2. Growth curves of different strains.** Wt: the wild type *M. abscessus*; ∆2362: *MAB_2362* knockout strain; C∆2362: *MAB_2362* complemented strain.


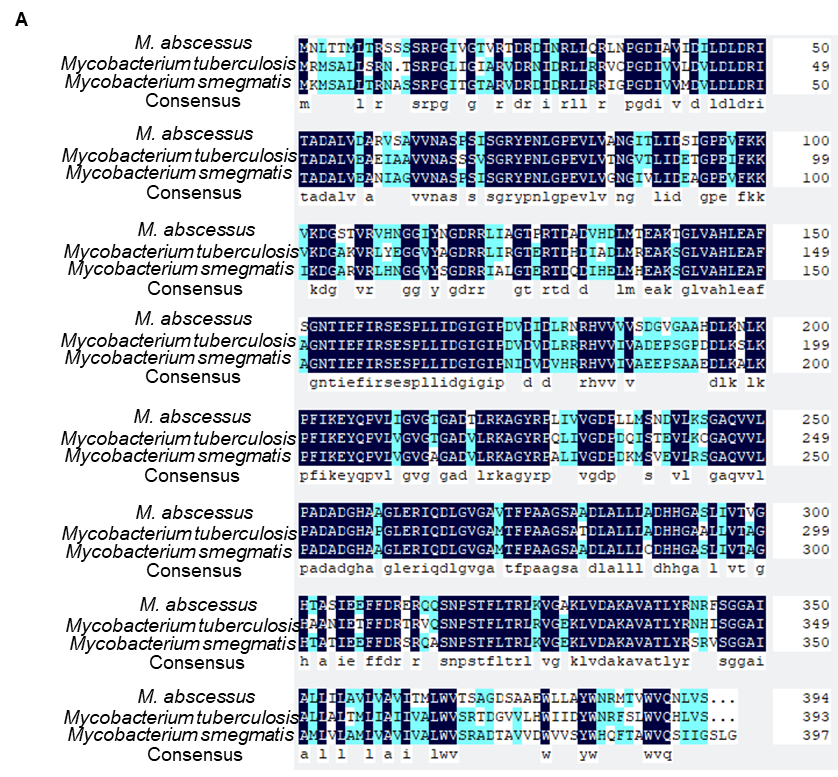


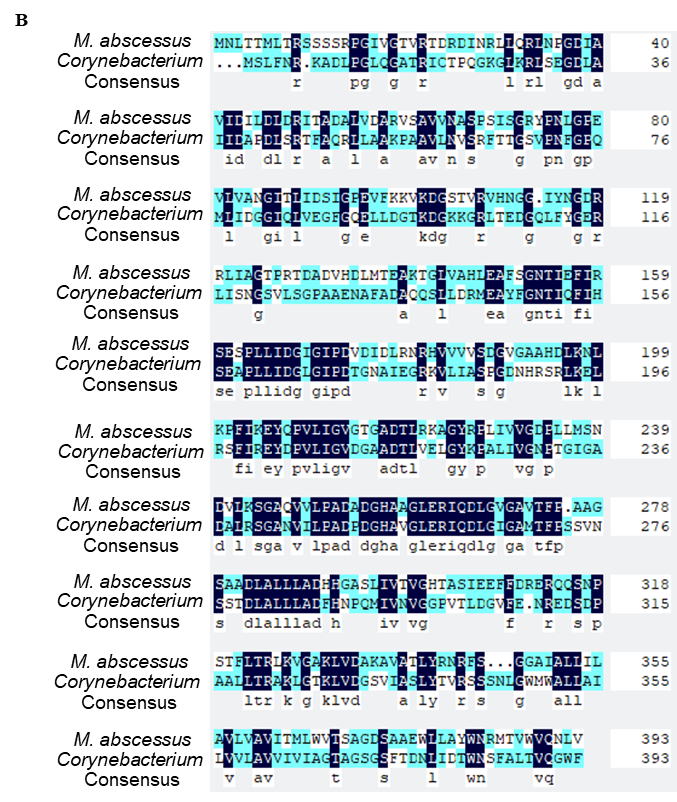
**Figure S3. Amino acid sequence alignments between MAB_2362 and homologs from different species.** (A) Comparison of the amino acid sequence of MAB_2362 with its homologous proteins in *M. tuberculosis* (Identity 72.59%) and *M. smegmatis* (Identity 74.81%). (B) The alignment of the amino acid sequence of MAB_2362 with the SteA amino acid sequence of *Corynebacterium glutamicum* (Identity 41.35%).


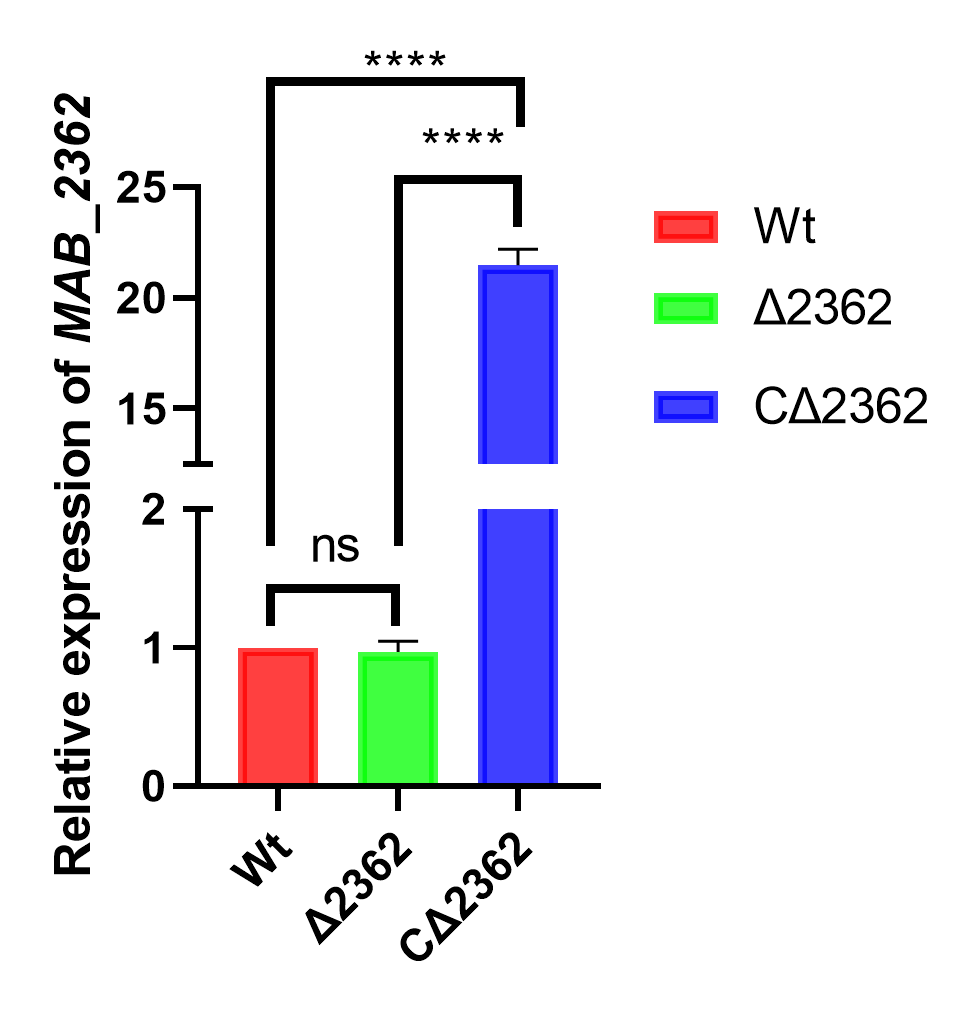
**Figure S4. Transcriptional expression levels of *MAB_2362* in Mab^Wt^, Mab^Δ2362^ and Mab^CΔ2362^ strains.** *MAB_2362* mRNA expression levels in Mab^Δ2362^, Mab^CΔ2362^ and Mab^Wt^ were measured by qRT-PCR by normalization with *sigA, a* housekeeping gene. ^****^, *P* < 0.0001. Wt: the wild type *M. abscessus*; Δ2362: *MAB_2362* knockout strain; CΔ2362: *MAB_2362* complemented strain.

**Table S4. MICs of various antibiotics against different *M. abscessus* strains**

|  | **Strains/MICs (µg/mL)^a^** | | | |
| --- | --- | --- | --- | --- |
| **Antibiotics** | **Mab^Wt^** | **Mab^Δ2362^** | **Mab^CΔ2362^** | **Mab^CΔ2362+2363^** |
| RIF | 64 | 2 | 16 | 16 |
| RFB | 2 | 0.25 | 1 | 1 |
| CLR | 32 | 2 | 8 | 8 |

^a^ Broth microdilution method was used to determine the MICs. The experiment was performed in triplicate and repeated twice.

RIF: Rifampicin; RFB: Rifabutin; CLR: Clarithromycin.

Mab^Wt^: the wild type *M. abscessus*; Mab^Δ2362^: *MAB_2362* knockout strain; Mab^CΔ2362^: *MAB_2362* complemented strain; Mab^CΔ2362+2363^: *MAB_2362* and *MAB_2363* complemented strain.


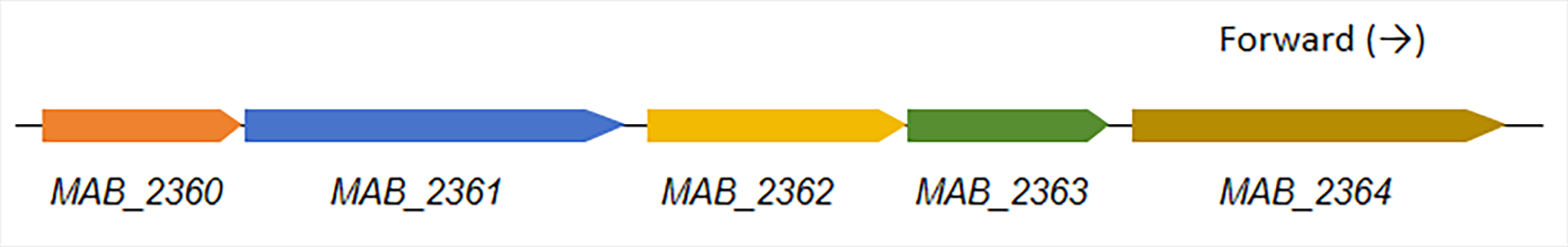
**Figure S5. Gene map of regions upstream and downstream of *MAB_2362* in *Mycobacterium abscessus* ATCC 19977.**
